# Erythromycin attenuates oxidative stress-induced cellular senescence *via* the PI3K-mTOR signaling pathway in chronic obstructive pulmonary disease

**DOI:** 10.3389/fphar.2022.1043474

**Published:** 2022-11-23

**Authors:** Yi Xiaofei, Li Tingting, Wei Xuan, He Zhiyi

**Affiliations:** Department of Respiratory and Critical Medicine, The First Affiliated Hospital of Guangxi Medical University, Nanning, Guangxi, China

**Keywords:** erythromycin, cellular senescence, oxidative stress, chronic obstructive pulmoanry disease, PI3K-mTOR signaling pathway

## Abstract

**Background and Purpose:** Chronic obstructive pulmonary disease (COPD) is proposed to hasten lung aging. Erythromycin protects against oxidative stress and inflammatory responses. However, the potential anti-senescence effect of erythromycin remains disclosed. In the present study, we investigated whether erythromycin influenced oxidative stress-induced cellular senescence and investigated its related mechanisms.

**Methods:** A cigarrete smoke (CS) -induced emphysema mouse model and a H_2_O_2_-induced premature senescence model in human bronchial epithelial cell line (BEAS-2B) were established. Senescence-related markers (P53, P21 and SA-β-Gal activity), and levels of oxidative stress biomarkers (MDA, SOD and ROS) were measured. Additionally, cells were pretreated with rapamycin (mTOR inhibitor) or erythromycin, and the expression levels of components of the PI3K-mTOR signaling pathway were measured in BEAS-2B cells.

**Results:** Exposed to H_2_O_2_, increased SA-β-gal activity was observed in BEAS-2B cells suggesting premature senescence. Erythromycin inhibited the expression of P53 and P21 in the CS-induced emphysema mouse model. MDA levels significantly increased and SOD levels decreased in the CS-exposed mice and H_2_O_2_-induced BEAS-2B cells. Rapamycin and erythromycin significantly suppressed the expression of P53 and P21. Additionally, rapamycin and erythromycin inhibited the PI3K-mTOR signaling pathway.

**Conclusion:** Our findings suggest that erythromycin ameliorates oxidative stress-induced cellular senescence *via* the PI3K-mTOR signaling pathway. Hence, we establish a theoretical foundation for the clinical application of erythromycin for COPD prevention and treatment.

## 1 Introduction

Chronic obstructive pulmonary disease (COPD) is an aging-related disease caused by chronic exogenous and endogenous oxidative stress (Barnes, 2017). Strikingly, cigarette smoke (CS) is responsible for 80% of COPD cases (Labaki and Rosenberg, 2020). Approximately 6,000 compounds and 10^15^ organic radicals are present in each puff of smoke, including reactive oxygen species (ROS) (Rahman I, 1996). Oxidant-induced lung inflammation and tissue damage ultimately induce a premature lung aging and cellular senescence. The bronchial epithelium, the main barrier between the external environment and the internal milieu, is one of the primary sources of inflammatory factors in COPD patients (Barnes and Donnelly, 2019; Cipollina et al., 2022). Upon aging, the bronchial epithelium repairs tissue more slowly, making the body more susceptible to external stress. This culminates in harmful feedback that deteriorates COPD. Thus, finding strategies to prevent cellular senescence of bronchial epithelium is a key for COPD treatment.

Aging is a process during which tissues and organs progressively lose their functions or do not function efficiently (Mathon NF, 2001; Kuilman et al., 2010). The cellular senescence process can be divided into replicative and stress-related senescence through P53 and P16INK4a activation, which activates P21 and induces cell cycle arrest (Kunlin, 2010). Currently, there are two main theories to explain aging: the programmed theory and the damage theory (Kunlin, 2010). As with the processes that govern child development, aging follows a biological timetable. People are essentially programmed to age. Meanwhile, the damage theory suggests that aging is caused by external or internal environmental aggressions, such as oxidative stress. Indeed, excessive intracellular ROS, leads to oxidative damage and increased inflammation (Harman, 1956), and is perceived as the most critical pathway of cellular senescence and body aging. The pathophysiology of COPD fits with the damage theory of aging. This is corroborated by Vij et al. (2018) and his colleagues, who have reported that senescence-associated markers P53, P21, and P16 are increased in CS-exposed BEAS-2B cells and mice, accompanied by autophagy impairment. Recently, our lab has also confirmed that CS could induce atrophy and senescence *in vitro* (Li et al., 2021). Thus, aging is implicated in COPD pathogenesis and its underlying pathways may unveil novel treatment targets.

The PI3K-mTOR signaling pathway is crucial for cellular senescence and aging, according to accumulating research (Barnes, 2017). It was shown that mTOR depletion extends lifespan in yeast, nematodes, flies, and mammals (Jia et al., 2004; Kapahi et al., 2004; Kaeberlein et al., 2005; Powers et al., 2006; Wu et al., 2013). Similarly, rapamycin treatment promotes longevity and health span in several age-related diseases (Johnson et al., 2013). The mTOR complex consists of two components, namely mTORC1 and mTORC2, in which rapamycin inhibits the first component. mTORC1 is activated by cellular stresses, such as increased oxidant generation. Indeed, Houssaini et al. (2018) and his colleagues have verified that mTOR activation could induce lung aging, accelerate inflammation and lung emphysema. This opens the possibility that blocking the mTOR pathway to inhibit cellular senescence can develop into a promising therapeutic strategy for COPD patients.

Macrolides are a group of antibiotics isolated from *streptomyces* that include rapamycin, azithromycin and erythromycin. Numerous clinical trials have shown that macrolides are beneficial to treat inflammatory airway diseases, including COPD. Our previous studies have demonstrated that long-term and low-dose erythromycin (125 mg, 3 times/day) is able to reduce frequency of COPD exacerbations and inhibit airway neutrophilic inflammatory responses (He et al., 2010; Cameron et al., 2012; Spagnolo et al., 2013). The GOLD 2020 and other clinical guidelines have recommended erythromycin as a treatment for repeated acute exacerbations to improve patients’ life quality (Criner et al., 2015; Halpin et al., 2021). Our lab has also reported that erythromycin inhibits CS-induced inflammatory responses *via* the PPARγ/NF-κB pathway (Qiu et al., 2021) and reverses the decreased expression of HDAC2 by regulating the JNK/c-Jun pathway (Bin et al., 2020). It appears that erythromycin may target multiple pathways to exert its anti-inflammatory effects, possibly alleviating the progression of COPD. However, it is unclear if erythromycin exerts anti-senescence effects in this setting.

In this study, we first established a CS-induced emphysema mouse model to determine whether erythromycin could protect against oxidative stress and lung aging. Next, we investigated whether erythromycin could alleviate oxidative stress-induced cellular senescence through the PI3K-mTOR signal pathway *in vitro*.

## 2 Materials and methods

### 2.1 CS-induced emphysema mouse model

Twenty-four male C57BL/6J mice (6–8 weeks old, 20–25 g) were purchased from the Guangxi Medical University Animal Center (Nanning, China). We randomly divided mice into three groups: Air (n = 8), CS (n = 8) and erythromycin (EM) (n = 8) groups. C57BL/6J mice were either exposed to room air or CS for 6 months. The CS-induced emphysema mouse model was based on our previous work (Zhang et al., 2019; Li et al., 2021). In brief, the CS group was exposed to five cigarettes (Nanning Zhenlong unfiltered cigarettes: 10 mg of tar and 0.9 mg of nicotine) for 30 min each time, including 30-min smoke-free intervals (4 times/day, 5 days/week, 6 months). The EM group was gavaged with injecting EM (100 mg kg^−1^/day, Sigma Aldrich) before the CS exposure. Mice were anesthetized and terminated by administering sodium pentobarbital (40 mg/kg, i. p.). Animal experiments were performed according to the protocols approved by the Animal Research of Guangxi Medical University Ethics Committee.

### 2.2 Lung histopathology and immunohistochemistry

The lung tissue was fixed in 10% formalin, embedded and sectioned (4 μm) for hematoxylin and eosin (HE) and immunohistochemistry (IHC). Lung tissue sections were dewaxed with xylene, hydrated with ethanol, and sequentially stained with HE. Images were taken from five different fields using a fluorescence pathological microscope (Olympus */BX53 + DP80, Japan), and Image-Pro Plus software 6.0 (Media Cybernetics) was used to measure the mean linear intercept (MLI) for each group, as an estimate of alveolar diameter.

The expression levels of P53 and P21 were determined by IHC. Briefly, lung tissue sections were deparaffinized with xylene, hydrated with alcohol, repaired with EDTA and then incubated with antibody against mouse p53 (Abclone, Wuhan, China) and p21 (Abclone, Wuhan, China) overnight at 4°C. On the following day, the sections were incubated with secondary Goat Anti-Rabbit IgG H&L (HRP) antibody (Abcam, Cambridge, UK) and stained with diaminobenzidine (Solarbio, DA1010, China). Images of lung sections were captured and analyzed as described above.

### 2.3 Cell culture and treatment

The BEAS-2B cell was purchased from the Cell Bank of the Chinese Academy of Sciences (Shanghai, China), and was cultured in DMEM medium (Gibco, Shanghai, China) supplemented with 10% fetal bovine serum (FBS, BI, Israel), 1% penicillin/streptomycin and kept at 37°C and 5% CO_2_, until the confluence reached 75%. Cultured cells were treated with H_2_O_2_ and erythromycin (Sigma Aldrich) or rapamycin (Abmole, United States). BEAS-2B cells were exposed to 100 μM H_2_O_2_ for 2 h, then washed twice with PBS and re-incubated with erythromycin or rapamycin. This treatment regimen was continued for three consecutive days. After that, the media was removed, wells were washed twice with PBS, and cells were harvested. The H_2_O_2_ concentration was chosen based on senescence induction assessed by the CCK8 proliferation assay.

### 2.4 Cell viability assay (CCK8 assay)

5 × 10^3^ cells/well were planted onto 96-well culture plates. After indicated treatments, the cell viability was evaluated using a cell counting kit-8 (CCK-8, Dojindo, Japan) and analyzed by measuring the absorbance at 450 nm with a microplate reader (BioTek, Florida, United States).

### 2.5 Oxidative stress assays

The homogenate of mouse lung tissue or prepared BEAS-2B cells were harvested and the following tests were performed.

#### 2.5.1 Measurement of ROS

The non-fluorescent probe 2′,7′-dichlorodihydrofluorescein diacetate (DCFH2-DA, Beyotiome, Shanghai, China) was used to quantify total ROS. Cells were plated in a 6-well plate, washed twice with PBS and co-incubated for 25 min at 37°C in the dark with serum-free growth media containing 10 μM DCFH-DA. Following the H_2_O_2_ incubation, cells were washed twice with medium. We used a BioTek Cytation five fluorescence microscope to determine the mean fluorescence intensity.

#### 2.5.2 ELISA assay for SOD and MDA

Superoxide dismutase (SOD) and malondialdehyde (MDA) levels in mouse lung tissue homogenate and cell culture media were evaluated using an ELISA kit according to the manufacturer’s procedure (Cloud-Clone Corp, Wuhan, China). A microplate analyzer was used to measure the absorbance at 450 nm (BioTek, Florida, United States).

### 2.6 SA-β-gal activity assay

A total of 1 × 10^4^ cells/well were cultivated in 24-well culture plates. Stimulated cells were stained with SA-β-gal according to manufacturer’s instructions (GENMED, Shanghai, China), and positive cells (blue cells) were captured using an optical microscope (Olympus, Japan). Five different fields per group were measured and the ImageJ software was used to determine the percentage of positive cells.

### 2.7 Protein isolation and western blotting

Total proteins were isolated from lung tissues and BEAS-2B cells. Samples were washed three times with PBS and lysed using RIPA buffer (Solarbio, Beijing, China). A total of 50 μg protein was loaded onto 8% or 12% SDS-PAGE and electro-transferred onto PVDF membranes. For blocking, membranes were incubated with 5% skim milk for 1 h. This was followed by incubation of the membranes with primary antibodies against p-mTOR, mTOR, PI3K, p-AKT, and AKT (1:1,000, CST, United States), p53 (1:800, Abclone, China), and p21 (1:500,Abclone, China) overnight at 4°C. GAPDH (1:2,000, SAB, United States) was used as the loading control. After incubation with secondary antibodies (1:20,000, CST, United States) for 1 h, membranes were analyzed using an Odyssey Imaging System. The signal was quantified with ImageJ software.

### 2.8 RNA isolation and Real Time-PCR

RNA was extracted from lung tissues using TRIzol (Sangon, Shanghai, China), and from BEAS-2B cells using RNA-easy (Vazmye, Nanjing, China). We prepared cDNA using the HiScript@III RT SuperMix for qPCR (Vazmye, Nanjing, China). Real Time-PCR was performed with 2x Q3 SYBR qPCR Master mix (TOLOBIO, Shanghai, China). We followed the manufacturer’s instructions and calculated the relative change in target gene expression using the 2^−△△Ct^ method. The PCR primer sequences were synthesized by Sangon Biotech Company and are shown in Table1.

**TABLE 1:** Primers used for the real Time-PCR.

P21-human	forward	GCC​CGT​GAG​CGA​TGG​AAC​TTC
	Reverse	CCT​GCC​TCC​TCC​CAA​CTC​ATC​C
P53-human	forward	GCC​CAT​CCT​CAC​CAT​CAT​CAC​AC
	Reverse	GCA​CAA​ACA​CGC​ACC​TCA​AAG​C
GAPDH-human	forward	CAG​GAG​GCA​TTG​CTG​ATG​AT
	Reverse	GAAGGCTGGGGCTCATTT
P21-mice	forward	ATG​TCC​AAT​CCT​GGT​GAT​GTC
	reverse	GAA​GTC​AAA​GTT​CCA​CCG​TTC
P53- mice	forward	TGG​AAG​GAA​ATT​TGT​ATC​CCG​A
	reverse	GTG​GAT​GGT​GGT​ATA​CTC​AGA​G
GAPDH- mice	forward	TGT​GTC​CGT​CGT​GGA​TCT​GA
	reverse	TTG​CTG​TTG​AAG​TCG​CAG​GAG

### 2.9 Statistical analysis

The SPSS 23.0 software was used for statistical analyses (IBM, New York, United States). The experimental results were represented in terms of mean ± standard deviation (SD). ANOVA followed by the least significant difference (LSD) test, were used for multiple comparisons. GraphPad Prism8 (GraphPad Software, Inc, La Jolla, CA, United States) was used to generate graphs. A statistically significant difference was considered when *p < 0.05*.

## 3 Results

### 3.1 Erythromycin attenuated aging in a mouse model of CS-induced emphysema

**FIGURE 1:**
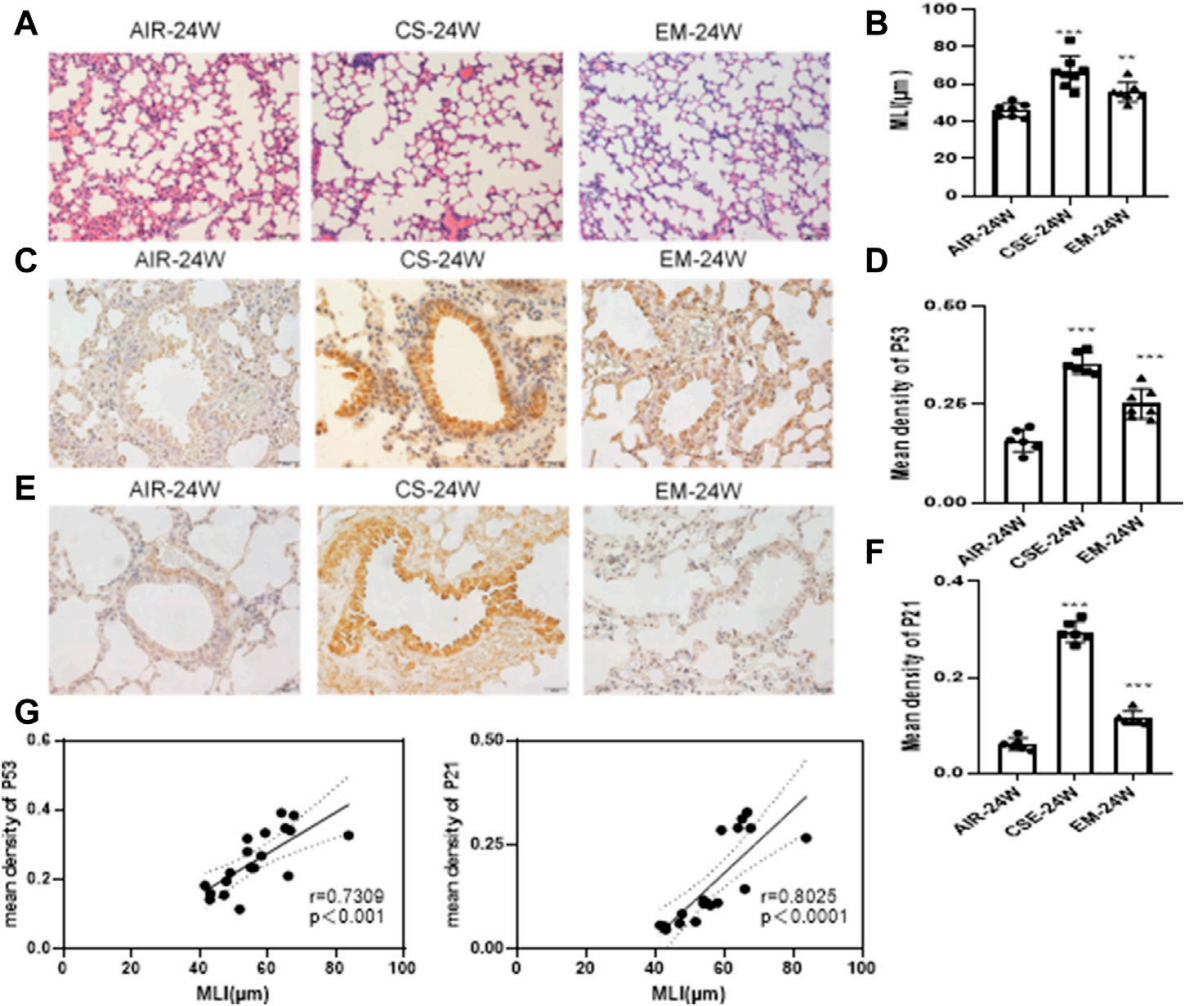
MLI and IHC staining of P53 and P21 in murine lungs **(A–B)** Alveolar MLI. Magnification: ×200. **(C–D)** IHC staining of P53. Magnification: ×400. **(E–F)** IHC staining of P21. Magnification:×400. **(G)** Scatter plot of correlation analysis for mean density of P53 and P21 staining with MLI in murine lungs. ***p < 0.0001. **p < 0.001. CS, cigarrete smoke. EM,erythromycin. MLI, mean linear intercept. IHC, immunohistochemistry.

To examine the influence of erythromycin on the pathogenesis of emphysema, a CS-induced emphysema mouse model was established. First, we evaluated the MLI to ensure that mice developed emphysema upon CS exposure. Indeed, the MLI of mice exposed to CS was significantly higher than those exposed to air (*p* < *0.05*). Alveolar expansion was still present, but the thickness of alveolar walls was lower than that of the control group (*p* < *0.05*) (Figures 1A,B). As shown in Figures 1C,E, IHC staining of P53 and P21 were increased in CS-exposed mice when compared to those of control ones (*p* < *0.05*). These proteins were mainly expressed in bronchial epithelial cells, supporting our decision to work with BEAS-2B cells in the following assays. Furthermore, the mean density levels of P53 and P21 were positively correlated with MLI (Figure 1G).

**FIGURE 2:**
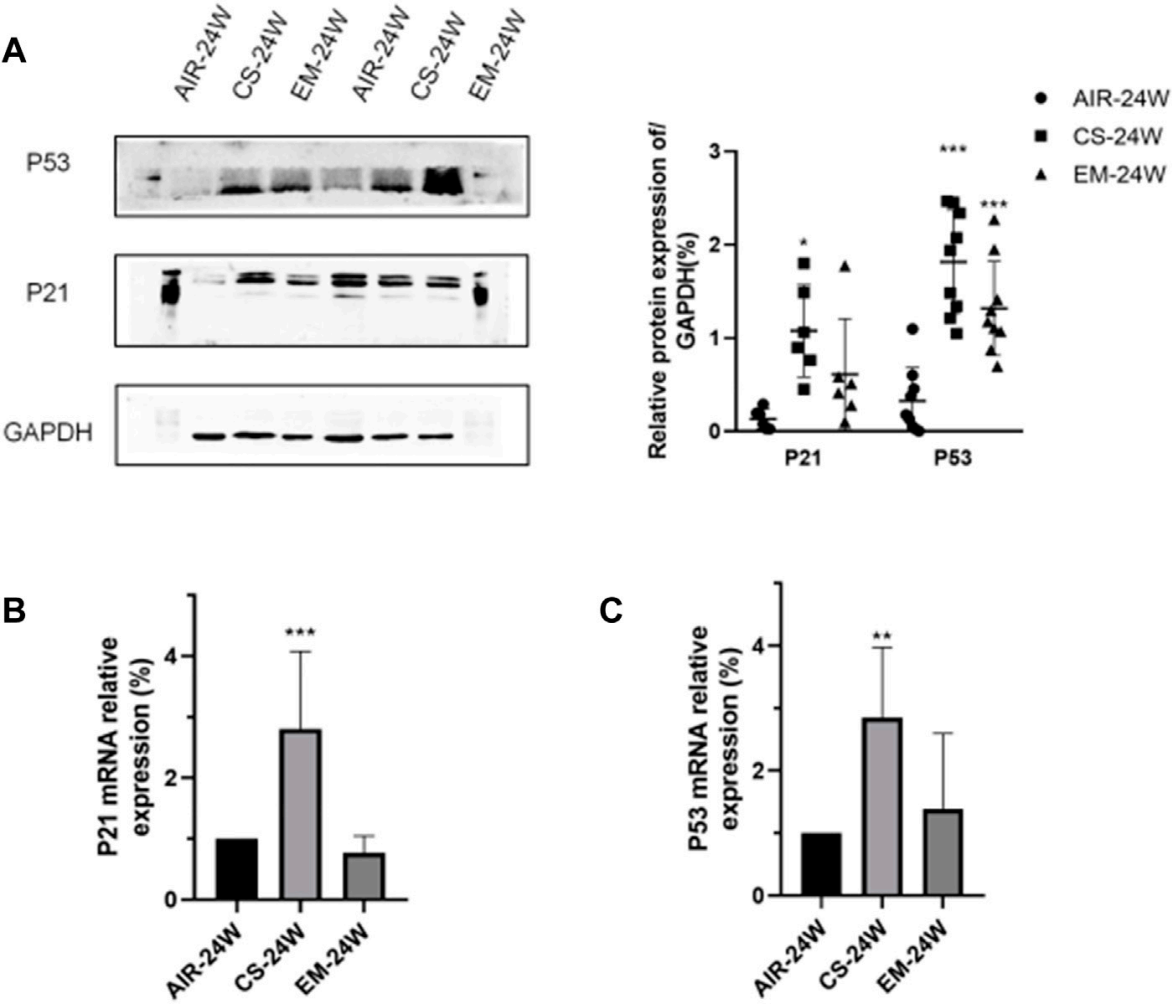
Erythromycin inhibited the expression of P53 and P21 in mice with CS-induced emphysema. **(A)**Protein expression levels of P53 and P21. **(B‐C)** mRNA expression levels of P53 and P21. ***p < 0.0001, **p < 0.001.

As shown in Figures 2A,B, in accordance with IHC results, both protein and mRNA expressions of P53 and P21 were higher in CS-exposed mice, while erythromycin treatment reduced the expression levels of P53 and P21 (*p* < *0.05*). Altogether, these results demonstrated that erythromycin treatment might prevents the rise of senescence markers P53 and P21 induced by chronic CS exposure.

### 3.2 Erythromycin inhibited oxidative stress in CS-exposed mice and H_2_O_2_-treated BEAS-2B cells

**FIGURE 3:**
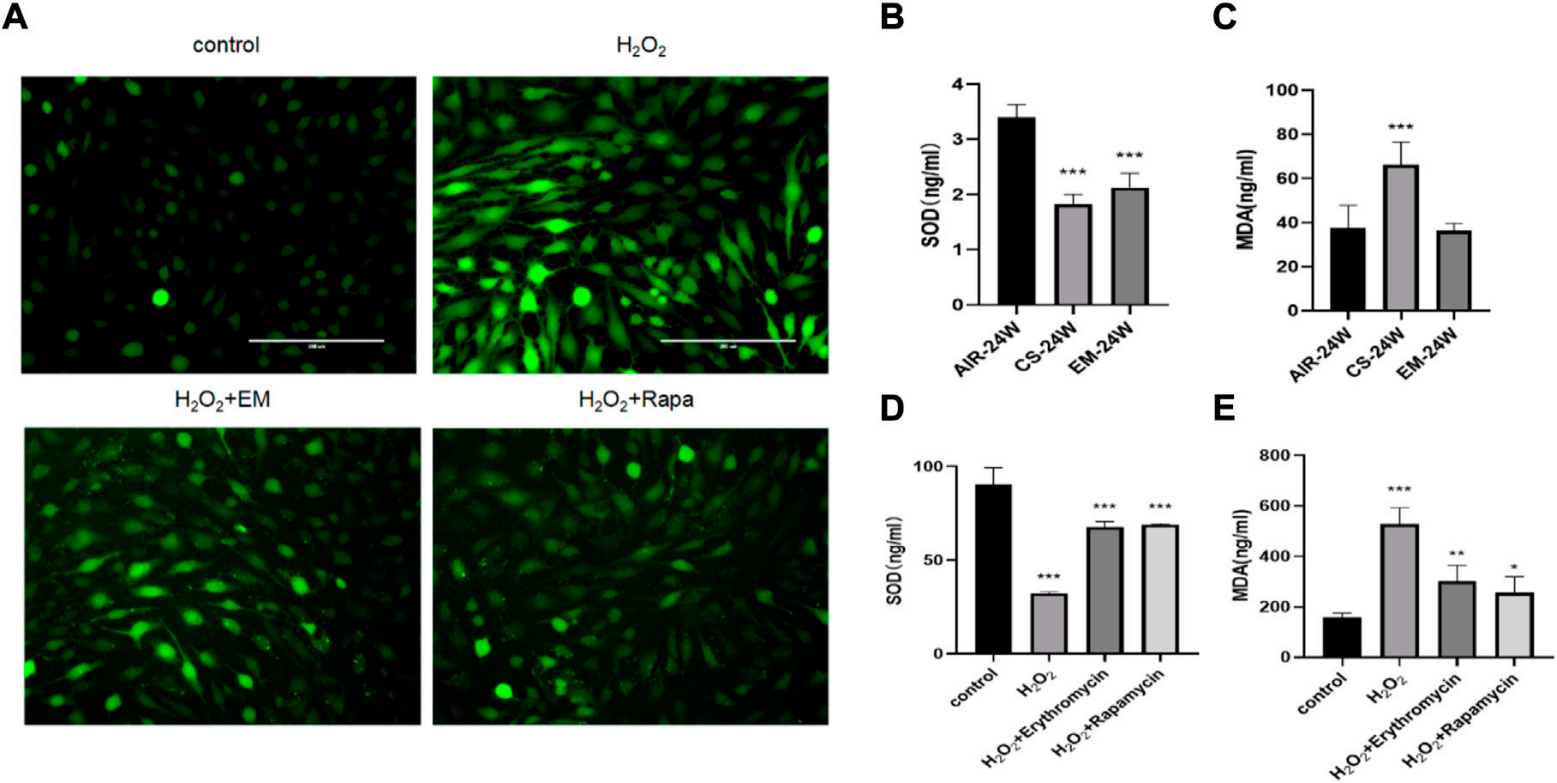
Antioxidant effect of erythromycin *in vivo* and *in vitro*. **(A)** Fluorescence photomicrographs of different groups of BEAS-2B cells. BEAS-2B cells were treated with 100 μ M H_2_0_2_,10 mg/m1 erythromycin or lOnM rapamycin. **(B–C)** SOD and MDA levels in mice. **(D–E)** SOD and MDA levels in BEAS-2B cells. ***p < 0.0001, **p < 0.001, *p < 0.05. EM, erythromycin. Rapa, rapamycin.

Next, we investigated the potential antioxidant effect of erythromycin on CS-exposed mice and BEAS-2B cells. Erythromycin and rapamycin were shown to decreased ROS production in H_2_O_2_-induced BEAS-2B cells. Compared with their respective control groups, the MDA content was significantly increased and SOD levels was suppressed in CS-exposed mice and of H_2_O_2_-treated BEAS-2B (*p* < 0.0*5*) (Figure 3). Therefore, erythromycin was able to reduce oxidative stress marker possibly through augmenting a cellular antioxidant system (SOD) in mice and BEAS-2B cells.

### 3.3 H_2_O_2_ induced cellular senescence in BEAS-2B cells

**FIGURE 4:**
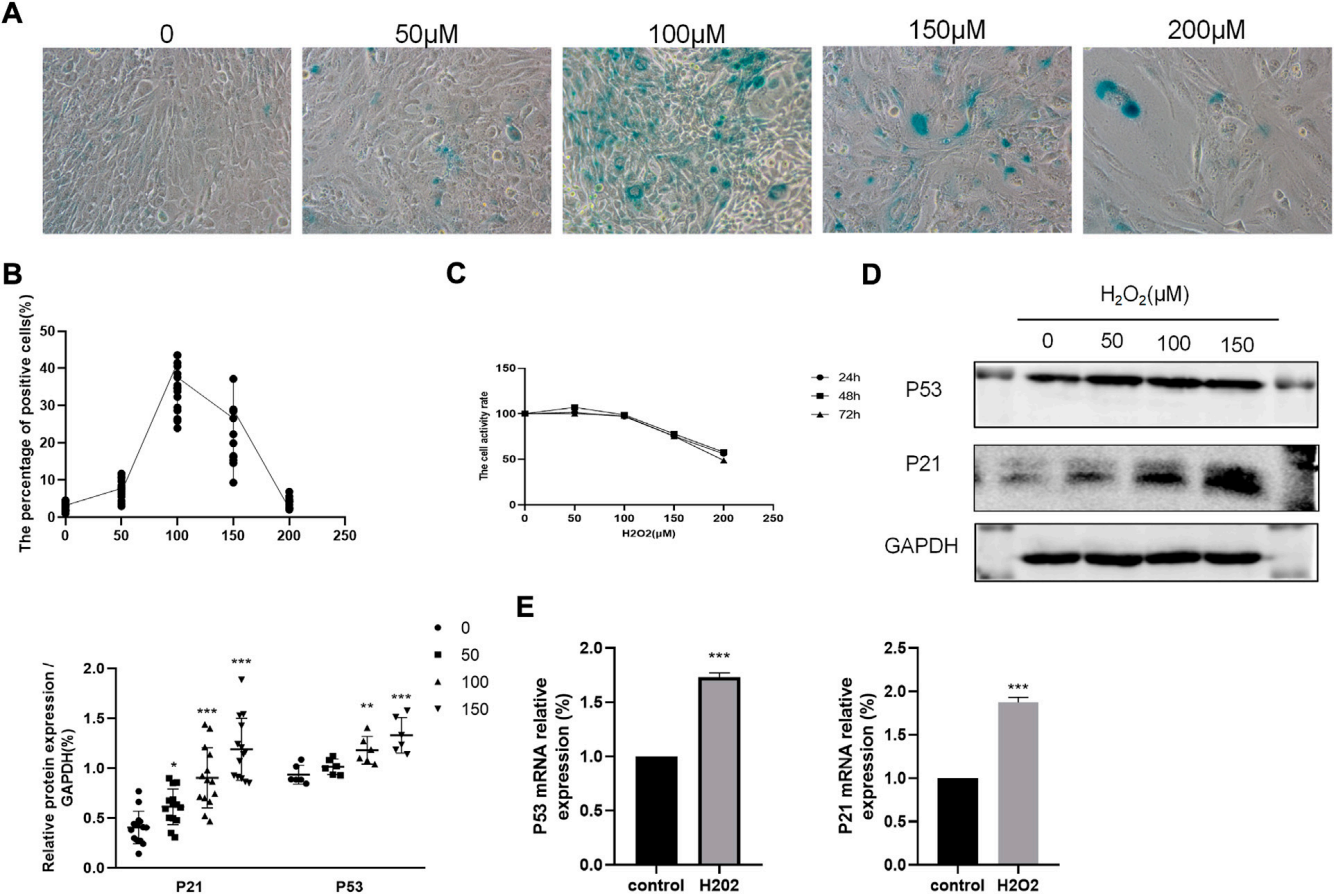
Effects of H_2_0_2_ in BEAS-2B cells. **(A–B)** SA- β -gal staining of BEAS-2B cells treated with different concentration of H_2_0_2_. Magnification: ×200. **(C)** Cell viability assay of BEAS-2B cells treated with different concentration of H_2_0_2_. **(D–E)** Effects of H_2_0_2_ on P53 and P21 protein and mRNA expression. ***p < 0.0001, **p < 0.001.

H_2_O_2_ has been reported to stimulate cellular senescence and oxidative stress, thus contributing to cell dysfunction. Based on cell proliferation assays, 100 μM H_2_O_2_ was used to induce cellular senescence (Figure 4C). Cellular senescence was determined by the SA-β-gal activity assay staining, in which aged cells were stained blue. In accordance with the cell proliferation assay, 100 μM H_2_O_2_ greatly increased the number of SA-β-gal positive cells (Figures 4A,B). In addition, compared to the control group, H_2_O_2_ treatment could dose-dependently increase the protein expression of P53 and P21 (Figure 4D). P53 and P21 mRNA levels were also elevated in H_2_O_2_ -treated cells (Figure 4E).

### 3.4 Erythromycin protected BEAS-2B cells against H_2_O_2_-induced cellular senescence

**FIGURE 5:**
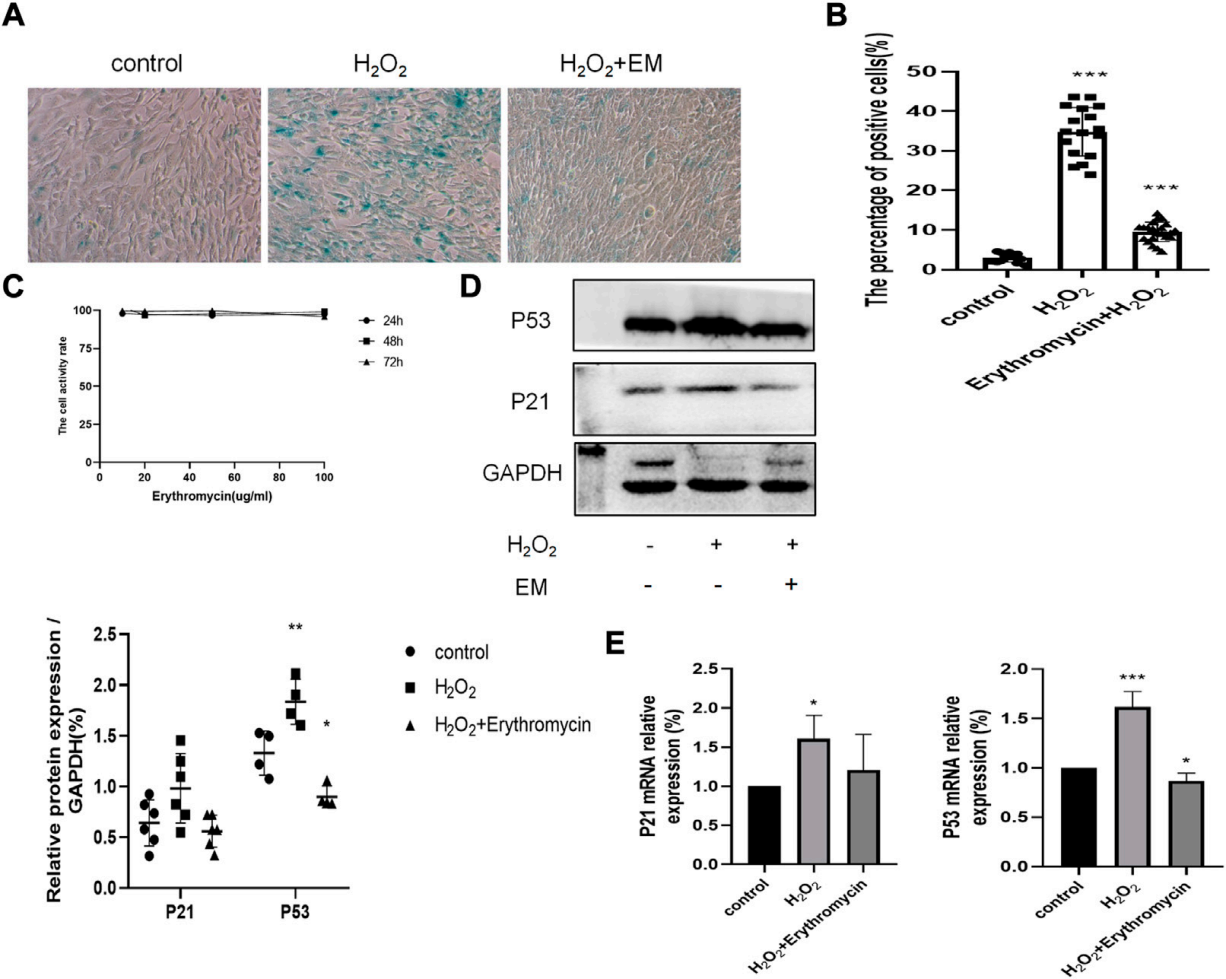
Effects of erythromycin in H_2_0_2_-treated BEAS-2B cells. **(A–B)** SA-13-gal staining of BEAS-2B cells treated with or without erythromycin and H_2_0_2_. Magnification: ×200. **(C)** Cell viability assay of BEAS-2B cells treated with different concentrations of erythromycin. **(D–E)** Expression of senescence markers P53 and P21. ***p < 0.0001, **p < 0.001,*p < 0.005.

We first established that up to 100 μg/ml erythromycin had no obvious impact on cell viability (Figure 5C). Next, we pretreated BEAS-2B cells with 10 μg/ml erythromycin, and then exposed them to 100 μM H_2_O_2_ for the indicated time to assess cellular senescence and the expression of senescence-associated markers. Erythromycin alleviated the deleterious effects of H_2_O_2_ as shown by the reduction of the percentage of SA-β-gal positive cells (Figures 5A,B). Consistent with these data, the expressions of P53 and P21 were significantly reduced in cells treated with erythromycin, as shown in Figures 5D,E.

### 3.5 Erythromycin attenuated H_2_O_2_-induced cellular senescence by inhibiting the PI3K-mTOR signaling pathway

**FIGURE 6:**
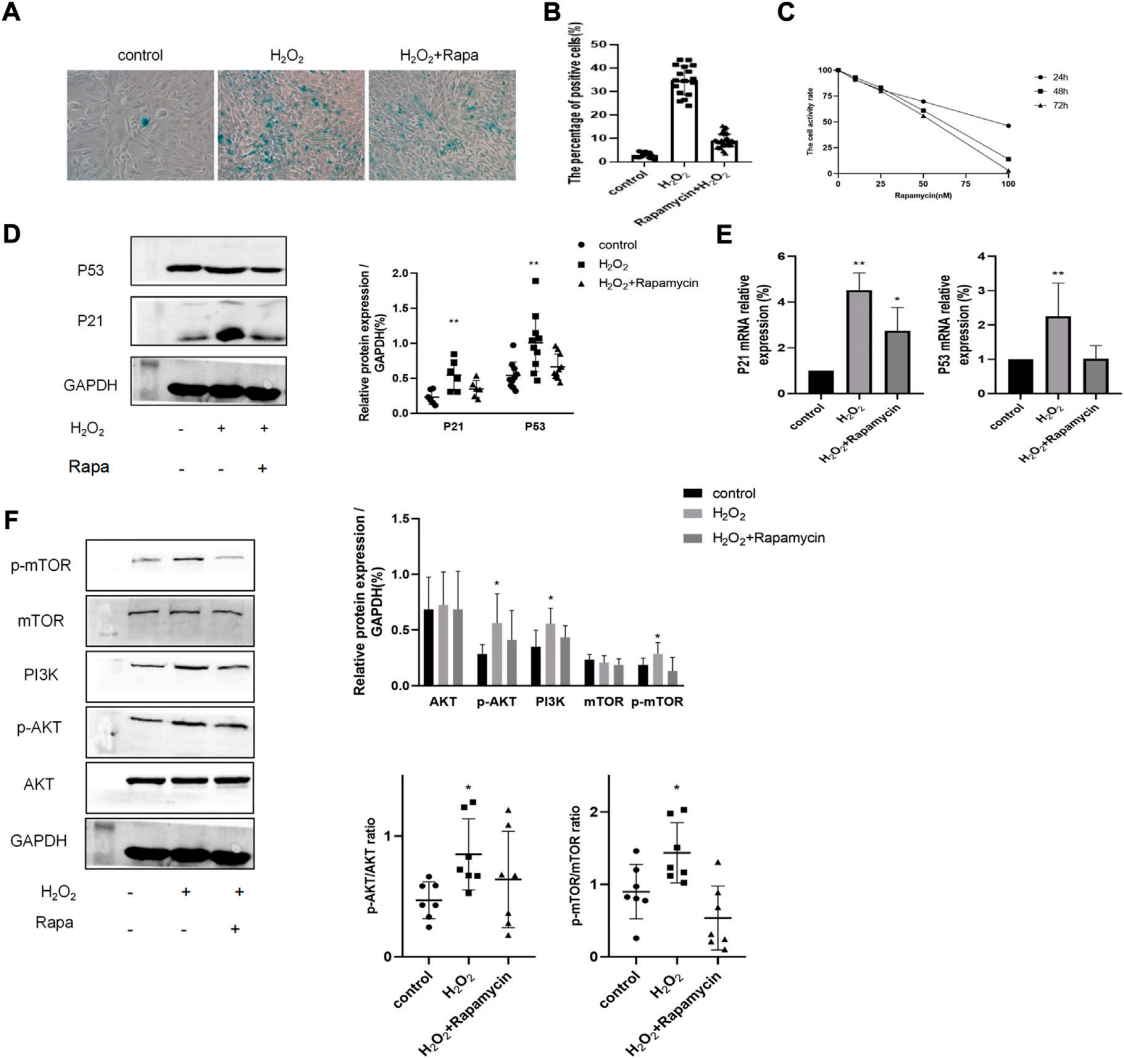
The PI3K-mTOR signaling pathway contributed to H_2_0_2_-induced cellular senescence in BEAS-2B cells. **(A–B)** SA-13-gal staining of BEAS-2B cells treated with or without rapamycin and H202. Magnification: ×200. **(C)** Cell viability assay of BEAS-2B cells treated with different concentrations of rapamycin. **(D–E)** Protein and mRNA levels of P53 and P21. **(F)** The expression level of proteins of the PI3K-mTOR pathway. **p < 0.001, *p < 0.005.

Next, we aimed to determine whether the PI3K-mTOR signaling pathway was activated by H_2_O_2_ in BEAS-2B cells. BEAS-2B cells were exposed to 100 μM H_2_O_2_ for the indicated time and pretreated with 10 nM rapamycin, which is a well-established inhibitor of the PI3K-mTOR signaling pathway. Rapamycin concentration was based on results from the cell viability assay (Figure 6C). Then, we examined the SA-β-gal activity and expression of senescence-associated markers. It was clear that the percentage of SA-β-gal positive cells was decreased in rapamycin-treated cells, when compared with that of the H_2_O_2_ group (Figures 6A,B). Moreover, as shown in Figures 6D,E, rapamycin significantly reduced H_2_O_2_-induced cellular senescence since the expressions of P53 and P21 were both significantly decreased. Importantly, through western blotting experiments, we determined that the PI3K-mTOR signaling pathway was strongly activated (*p* < 0.0*5*) in H_2_O_2_-treated cells (Figure 6F).

**FIGURE 7:**
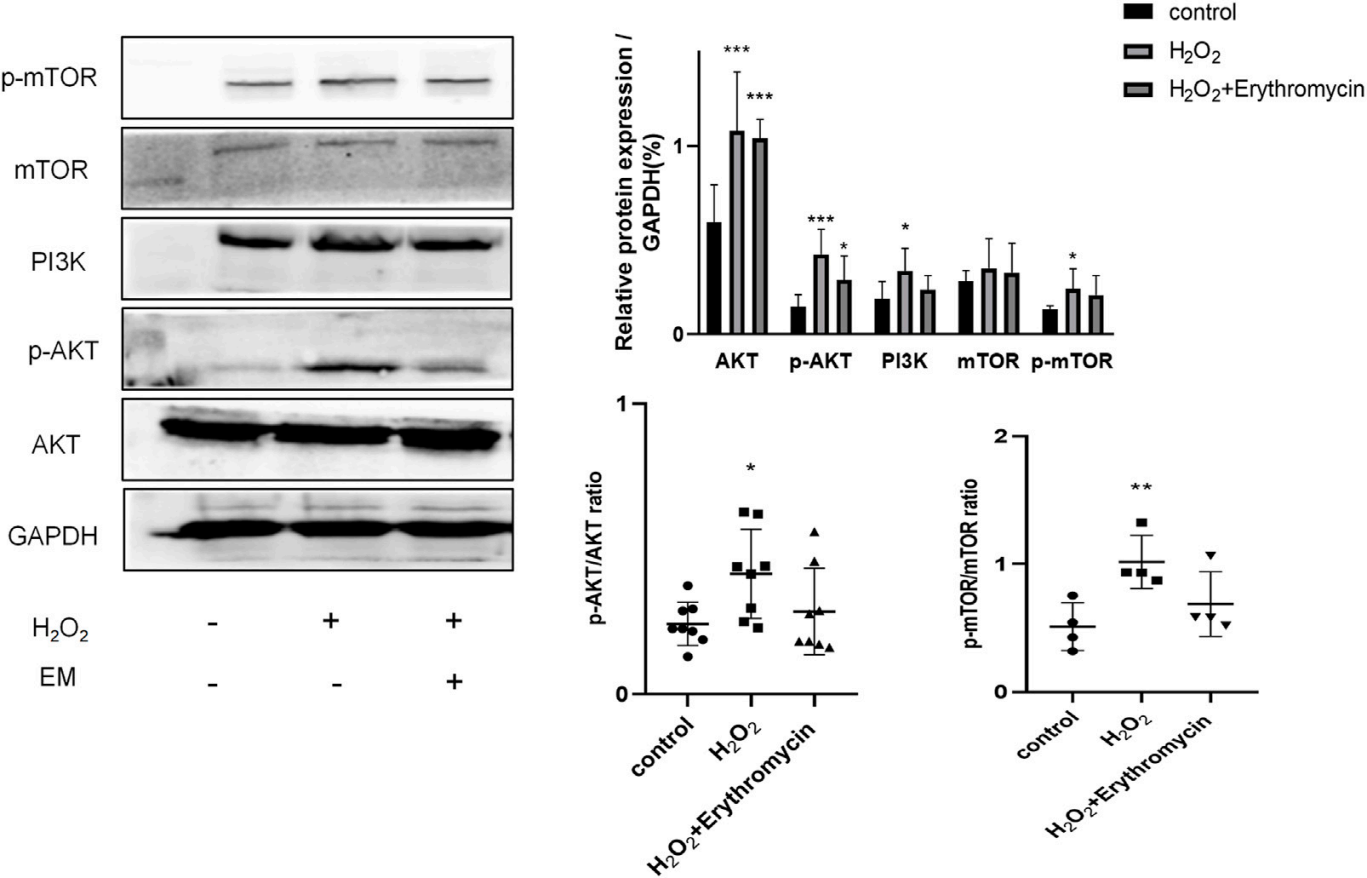
Erythromycin inhibited H_2_0_2_-induced BEAS-2B cellular senescence *via* the PI3K-mTOR signaling pathway. The expression level of proteins of the PI3K-mTOR pathway was assessed using western blot. ***p < 0.0001, **p < 0.001,*p < 0.005.

Next, we investigated whether erythromycin, like rapamycin, could attenuate cellular senescence *via* the inhibition of the PI3K-mTOR pathway. Experimental data revealed that erythromycin remarkably inhibited the PI3K-mTOR signaling pathway, since there was a strong down-regulation of PI3K, p-AKT and p-mTOR protein levels (*p* < 0*.05*) (Figure 7).

## 4 Discussion

In this study, we investigate whether erythromycin inhibits oxidative stress and cellular senescence *in vitro* and *in vivo*. By referring to our previous research methods, we established a mouse model of chronic emphysema. 30 min before being exposed to cigarette smoke, mice in the erythromycin treatment group were gavaged with erythromycin solution. The current study showed that erythromycin alone had no significant effect on lung inflammation compared with the air group (Zhang et al., 2019), so no drug control group was set in this experiment. The up-regulation of aging-related markers P53 and P21 was the first thing we saw in a mouse model of emphysema brought on by prolonged cigarette smoking. Additionally, it was primarily expressed in bronchial epithelial cells, which suggested that the lung tissues of mice with emphysema had undergone senescence. And tobacco smoke is a complex mixture of more than 6,000 compounds and 10^15^ free radicals. We intended to further investigate the impact of erythromycin on COPD *in vitro* experiment from the standpoint of oxidative stress. Hydrogen peroxide is an oxidant molecule that readily permeates cell membranes. The oxidative damage generated by H_2_O_2_ can stimulate oxidative stress *in vivo* and this oxidant has been used to develop *in vitro* models (Xia et al., 2020; Cao et al., 2021; Mahajan et al., 2021; Ok et al., 2021). To investigate the anti-senescence effect of erythromycin, we used H_2_O_2_ to establish a premature senescence model in BEAS-2B cells. Our data corroborated that CS significantly increased senescence-associated markers as well as oxidative stress parameters. Similarly, there were significant pathological changes in the lungs of mice exposed to CS. We also found that the protein expression of the PI3K-mTOR signaling pathway was dramatically increased in H_2_O_2_-treated BEAS-2B cells. Erythromycin inhibited cellular senescence and oxidative stress in H_2_O_2_-induced BEAS-2B cells by inhibiting the PI3K-mTOR signaling pathway. Using CS-exposed mice and H_2_O_2_-treated BEAS-2B cells, this study showed that erythromycin significantly reduced oxidative stress and cellular senescence *via* the PI3K-mTOR pathway.

COPD represents a major public health issue and has become the third leading cause of death in the world. Accumulating evidence has revealed that cellular senescence leads to COPD development (Cho and Stout-Delgado, 2020; Schneider et al., 2021). Indeed, oxidative stress is a driving mechanism of COPD senescence, and is the main feature of this disease (Barnes, 2020). There are several treatment strategies available for COPD patients, including smoking cessation, bronchodilator therapy, oxygen therapy, pulmonary rehabilitation, bronchoscopic lung volume reduction and lung transplantation (Fabbri et al., 2004). However, there is no effective strategy to reverse the decrease in lung function that these patients experience. Therefore, COPD treatments can alleviate symptoms and enhance life quality without affecting disease progression. The discovery of novel COPD treatments is still a pressing issue.

Smoking-induced oxidative stress is an important factor in COPD development (Liguori et al., 2018; Luo et al., 2020). COPD patients have higher levels of biomarkers related to oxidative stress, such as 4-HNE, 8oxodG and MDA, all of which display a strong correlation with airflow limitation in elderly patients (Aggarwal et al., 2018; Liguori et al., 2018). When smoke is inhaled, it increases the generation of ROS and impairs the antioxidant defense system, causing damage to the lung tissue and cells (Barnes, 2020). SOD is part of a system of antioxidant enzymes that remove oxyradicals from the body. The SOD activity may translate into a good indicator of tissue damage. Meanwhile, MDA is an indicator of ROS production because oxidative stress damages cellular membranes and induces lipid peroxidation. Therefore, SOD and MDA are important parameters in estimating the level of oxidative stress. We found that CS exposure elevated the expression of P53 and P21 *in vivo*, which were accompanied by an increase in MDA levels and a decrease in SOD activity. The MLI of CS-exposed mice was significantly higher than that of air-exposed animals, while some alveoli were fused in CS-exposed mice (Figure 1A). After erythromycin treatment, indicators of oxidative stress and lung aging were reduced in CS-exposed mice. Therefore, these preliminary studies proved that erythromycin could reduce lung aging in CS-exposed mice, which was also confirmed *in vitro*. Interestingly, both P53 and P21 were mainly expressed in bronchial epithelial cells (Figures 1C–E). The bronchial epithelium is susceptible to damage from inhaled environmental pathogens and contaminants, which often promote senescence and activate associated pathways.

Next, we evaluated whether the PI3K-mTOR signal pathway was involved in the H_2_O_2_-induced cellular senescence. As known, the mTOR signaling pathway is involved in cell survival, growth, and metabolism (Sadighi Akha, 2018). Recent studies have shown that activation of the mTOR signaling pathway stimulates cellular senescence. summer and his colleagues confirm that the up-regulation of the mTORC1/PGC1 signaling axis leads to cellular senescence of the lung epithelium (Summer and Schriner, 2021). Mice with an over-expression of mTOR rapidly develop emphysema, pulmonary hypertension, and pulmonary inflammation, while low-dose rapamycin could inhibit mTOR expression, thereby inhibiting cellular senescence and inflammatory senescence-associated secretory phenotypes (Houssaini et al., 2018). Furthermore, mTOR is necessary for oxidative stress-induced cellular senescence, and that oxidative stress is associated with the mTOR activation in COPD patients (Iglesias-Bartolome et al., 2012). In COPD patients’ lung tissue and pulmonary artery smooth muscle cells, activation of the mTOR pathway is accompanied by up-regulation of aging marker proteins P16 and P21 expression (Houssaini et al., 2018). In parallel, our experimental results demonstrated that the activation of the PI3K-mTOR related pathway accompanied the increase in senescence in H_2_O_2_-stimulated BEAS-2B cells. As a potential new therapeutic target for COPD, the suppression of the mTOR signaling pathway to lessen cellular senescence is being considered. After treatment with rapamycin, which is a mTOR inhibitor, we discovered that the expression of PI3K-mTOR related pathway was down-regulated in BEAS-2B cells, in addition to β-galactosidase positive decreased expression and down-regulated expression of age-related genes P21 and P53. As a potential treatment for age-related diseases, rapamycin shows great promise (Johnson et al., 2013; Partridge et al., 2020). Clinically, rapamycin is broadly applied as an immunosuppressant for the treatment of organ transplant rejection and autoimmune diseases, such as lymphangiomyomatosis (LAM). However, the adverse effects of rapamycin limited its clinical application to some degree. Some of the side effects of rapamycin are oral ulcers, anemia, pneumonia, and wound healing. This somewhat restricts the use of rapamycin in the treatment of COPD.

By contrast, erythromycin, a macrolide like rapamycin, has been widely utilized in clinical treatment, and has been demonstrated to be helpful in COPD patients. Moreover, compared with rapamycin, erythromycin is inexpensive and readily available. The anti-inflammatory effect of erythromycin has been reported in several respiratory diseases. In PBMCs obtained from COPD patients and in U937 cells treated with CSE, erythromycin inhibits the JNK/c-Jun pathway to improve corticosteroid sensitivity (Bin et al., 2020). Additionally, erythromycin was found to reduce H_2_O_2_-induced oxidative stress and inhibit AP-1 activity in human bronchial epithelial cells (He et al., 2008). Based on previous reports, we sought to explore if erythromycin may reduce cellular senescence *via* the PI3K-mTOR pathway, hence slowing COPD development. Pretreated with erythromycin, H_2_O_2_-stimulated BEAS-2B cells down-regulated the expression of the PI3K-mTOR pathway and lowered the positive expression of β-galactosidase, indicating a decrease in the amount of senescent cells. In addition, the protein and mRNA expression of the senescence-related genes P21 and P53 were also down-regulated. These results suggest that erythromycin inhibits H_2_O_2_-induced cellular senescence of BEAS-2B cells *via* the PI3K-mTOR pathway, which may serve as a basis for the treatment of COPD. This phenomenon is similar to pretreatment with the mTOR specific inhibitor rapamycin.

However, our study has some limitations. *In vivo* and *in vitro*, over-expression of the pi3k-mTOR pathway is still required to confirm the role of erythromycin in preventing cellular senescence. Additionally, downstream molecules of the mTOR pathway, as well as P53-related acetylation, should be assessed in future studies. Meanwhile, the specific targets of erythromycin remain to be elucidated. Moreover, the long-term use of erythromycin can induce bacterial resistance and dysbiosis. Therefore, macrolide derivatives without antibacterial activity could be promising strategies for COPD treatment.

In conclusion, our preliminary research suggest that erythromycin protects against oxidative stress-induced cellular senescence and mediates anti-senescence effects in COPD. Studies have demonstrated that erythromycin slows down the aging process as well as the development of COPD. The development of novel treatments for COPD may be facilitated by this experimental study.

## Data Availability

The raw data supporting the conclusions of this article will be made available by the authors, without undue reservation.
